# Potential drug targets for myocardial infarction identified through Mendelian randomization analysis and Genetic colocalization

**DOI:** 10.1097/MD.0000000000036284

**Published:** 2023-12-08

**Authors:** Jiayu Wu, Qiaoming Fan, Qi He, Qian Zhong, Xianqiong Zhu, Huilian Cai, Xiaolin He, Ying Xu, Yuxuan Huang, Xingwei Di

**Affiliations:** a The First School of Clinical Medicine, Guangzhou University of Chinese Medicine, Guangzhou, China; b Clifford Hospital, Guangzhou University of Chinese Medicine, Guangzhou, China; c The Eighth Clinical College, Guangzhou University of Chinese Medicine, Guangzhou, China; d The First Affiliated Hospital of Jinzhou Medical University, China; e Shenzhen Clinical College, Guangzhou University of Chinese Medicine, China; f The Fourth Clinical Medical College, Guangzhou University of Chinese Medicine, Guangzhou, China; g School of Traditional Chinese Medicine, Guangdong Pharmaceutical University, Guangzhou, China.

**Keywords:** Drug target, genetic colocalization, Mendelian randomization, myocardial infarction

## Abstract

Myocardial infarction (MI) is a major cause of death and disability worldwide, but current treatments are limited by their invasiveness, side effects, and lack of efficacy. Novel drug targets for MI prevention are urgently needed. In this study, we used Mendelian randomization to identify potential therapeutic targets for MI using plasma protein quantitative trait loci as exposure variables and MI as the outcome variable. We further validated our findings using reverse causation analysis, Bayesian co-localization analysis, and external datasets. We also constructed a protein-protein interaction network to explore the relationships between the identified proteins and known MI targets. Our analysis revealed 2 proteins, LPA and APOA5, as potential drug targets for MI, with causal effects on MI risk confirmed by multiple lines of evidence. LPA and APOA5 are involved in lipid metabolism and interact with target proteins of current MI medications. We also found 4 other proteins, IL1RN, FN1, NT5C, and SEMA3C, that may have potential as drug targets but require further confirmation. Our study demonstrates the utility of Mendelian randomization and protein quantitative trait loci in discovering novel drug targets for complex diseases such as MI. It provides insights into the underlying mechanisms of MI pathology and treatment.

## 1. Introduction

Ischemic heart disease is an increasingly burdensome condition and one of the leading causes of death and disability in humans. The most harmful type of ischemic heart disease is acute myocardial infarction (MI) when the coronary arteries supplying blood to the heart become blocked due to plaque buildup or other reasons, leading to irreversible damage and necrosis of myocardial cells. In recent years, the incidence of MI has been on the rise, with a trend towards younger age at onset. Severe heart attacks can result in sudden death, accounting for 2-fifth of deaths in China.^[1]^ After treatment, MI can also lead to complications such as heart failure, arrhythmias, cardiac rupture, bleeding, and ischemia-reperfusion injury, resulting in serious adverse outcomes. Timely revascularization following MI, including percutaneous coronary intervention, thrombolysis, and bypass surgery, is crucial for improving heart function and preventing pathological remodeling after infarction.^[2]^ However, these effective yet invasive methods may not apply to all patients due to specific clinical characteristics. They may risk serious complications such as bleeding and reperfusion injury.^[2,3]^ Attempts to limit infarct size and improve prognosis through pharmacological treatments (such as antiplatelet agents, antiarrhythmic drugs, and angiotensin-converting enzyme inhibitors) without reperfusion have been proven generally ineffective due to non-targeted drug distribution, significant side effects, and short half-lives.^[4]^ Therefore, seeking more effective ways to protect myocardial cells, suppress cell death, alleviate inflammatory responses, and improve prognosis in the treatment of MI to avoid complications is imperative.

Mendelian randomization (MR) analysis has recently been widely utilized in drug target development and repositioning. For example, the correlation between ACE inhibitors in antihypertensive drugs and the risk of schizophrenia has been examined,^[5]^ and adrenomedullin has been identified as a promising target for treating heart failure.^[6]^ MR is a genetic instrumental variable analysis designed to evaluate causal relationships between exposures and outcomes, often using single nucleotide polymorphisms (SNPs) from genome-wide association studies (GWAS) as genetic instruments. MR screens drug targets by combining multiple independent common genetic variants within or near genes encoding proteins to create an instrumental variable miming a therapeutic intervention, providing direct evidence of a potentially causal relationship between the drug target and changes in exposure and outcome.^[7]^ Moreover, compared to observational studies, MR can mitigate the influence of confounding factors. MR can predict drug treatment targets and anticipate adverse reactions to these targets.

Protein quantitative trait loci (pQTL) refer to specific genomic regions or genetic variants that are associated with variations in the levels or expression of proteins in an organism. These genetic variants can influence the abundance of specific proteins within a given tissue or under certain conditions. The study of pQTL aims to identify and understand the genetic factors that contribute to the regulation of protein levels, thereby shedding light on the genetic basis of complex traits and diseases. Grundberg^[8]^ study explores the genetic regulation of protein levels across various tissues and provides insights into the genetic control of protein expression, shedding light on the concept of pQTL.

In this study, we employed MR to identify potential therapeutic targets for MI. Firstly, we utilized MR with recently published GWAS data for MI and plasma pQTL data from Zheng^[9]^ study to identify plasma proteins that may contribute to MI. Secondly, we conducted reverse causal analysis and Bayesian colocalization analysis to validate the initial findings further. Thirdly, we constructed an interaction network between the identified proteins and known MI targets. Finally, we repeated the analysis using different datasets for external validation to strengthen our conclusions.

## 2. Materials and methods

### 2.1. Plasma pQTL

For the primary analysis, we obtained plasma pQTL data from a study by Zheng et al,^[9]^ which integrated 5 previously published GWAS.^[10–14]^ To ensure the inclusion of relevant pQTLs, we applied specific criteria. These criteria were as follows:

1. We utilized a genome-wide significance threshold of *P* < 5 × 10 − 8 and a factor (F) value >10.
2. Loci within the major histocompatibility complex region (chr6, 26–34 Mb) were excluded.
3. We ensured independent association by applying low linkage disequilibrium clumping (r^2^ < 0.001).
4. We identified cis-acting pQTLs.

As a result, our analysis included 738 cis-acting SNPs corresponding to 734 proteins. To ensure the reliability of the data, we cross-referenced it with the original documents to verify its accuracy. In cases where essential information, such as the effect allele frequency, was missing in the QTL GWAS summary statistics, we completed the data using the matched human genome build as a reference.

### 2.2. GWAS summary statistics of MI

For our primary analysis, we utilized summary statistics from the most recently published GWAS dataset on MI.^[15]^ The dataset comprised 639,221 individuals of European ancestry, with 61,505 cases and 577,716 controls. For external validation, we obtained summary statistics from the CARDIoGRAMplusC4D study,^[16]^ which included 43,676 cases and 128,199 controls, and the FinnGen study,^[17]^ which included 12,801 cases and 187,840 controls.

### 2.3. MR analysis

In this study, we used plasma pQTLs as the exposure variable and MI as the outcome variable. We conducted MR analysis using the “TwoSampleMR” package available at [GitHub link]. When only 1 pQTL was available for a given protein, we applied the Wald ratio method. On the other hand, if multiple genetic instruments were available, we used the inverse variance-weighted MR approach.^[18]^ For the primary analysis, we established the *P* value threshold at .01. We performed MR analysis to externally validate the initially identified proteins using a *P* value threshold of .05. We used the same variant strategy and genetic instruments (SNPs) as those employed in the preliminary analysis to validate the initial results. Reverse causation analysis was conducted using the Steiger test.

### 2.4. Bayesian co-localization analysis

Bayesian co-localization analyses were performed to assess the likelihood of 2 traits sharing the same underlying causal variant. The “coloc” package available at [GitHub link] was utilized with default parameters for this purpose. As described previously, Bayesian co-localization yields posterior probabilities for 5 hypotheses related to a shared variant between the 2 traits. For this study, we examined the posterior probabilities of hypothesis 3 and hypothesis 4 (PPH4). Hypothesis 3 proposes that the protein and MI traits are associated with the region through distinct variants, while hypothesis 4 posits that both traits are linked to the region via shared variants. We used coloc.abf for the analysis. A gene was considered to show evidence of co-localization if its gene-based PPH4 surpassed 75%.

### 2.5. Relationship between MI-related proteins and cardiac risk factors

We obtained risk factors for heart disease from published GWAS summary studies and databases. These risk factors included arrhythmias, atrial fibrillation,^[17]^ body mass index, fasting blood glucose, high-density lipoprotein (HDL) cholesterol levels,^[19]^ low-density lipoprotein (LDL) cholesterol levels,^[20]^ MI,^[15]^ Type 2 diabetes,^[19]^ total cholesterol levels,^[21]^ and triglycerides (TG). The risk factors were used as outcome variables in our study.

**Figure 1.**
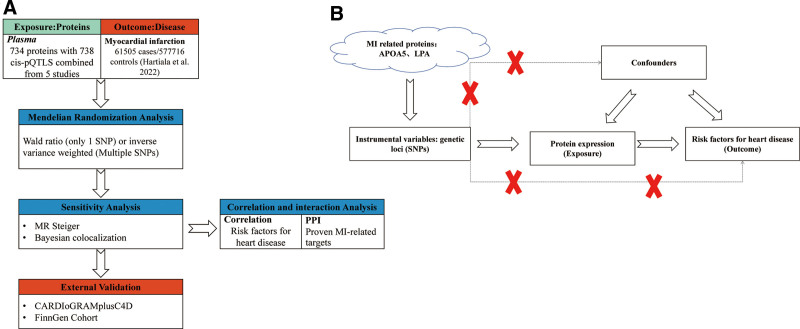
Flowchart and three hypotheses of MR. MR = Mendelian randomization.

Subsequently, we identified MI-related cis-pQTLs as exposure variables and SNPs as instrumental variables. Our study was based on 3 hypotheses (Fig. 1):

SNPs are strongly associated with risk factors.

SNPs are not associated with various confounding factors.

SNPs affect prognosis solely through their influence on the risk factors.

To conduct the study, we followed the STROBE-MR procedure.

### 2.6. Protein-protein interaction(PPI) networks and enrichment analysis

To investigate the interactions between MI-related targets and known disease targets, we searched “myocardial infarction” using the GeneCards database (https://www.genecards.org/). Targets with a Score > 20 were extracted. Subsequently, these targets were imported into the STRING database (https://string-db.org/), where the species was set as Homo sapiens, and the confidence level was set to high (0.700) in order to construct a PPI network. We further filtered the targets to include only those with direct evidence, which were then subjected to enrichment analysis using the “clusterProfiler”^[22]^ package.

## 3. Results

### 3.1. Screening the proteome for MI-causing proteins

**Figure 2.**
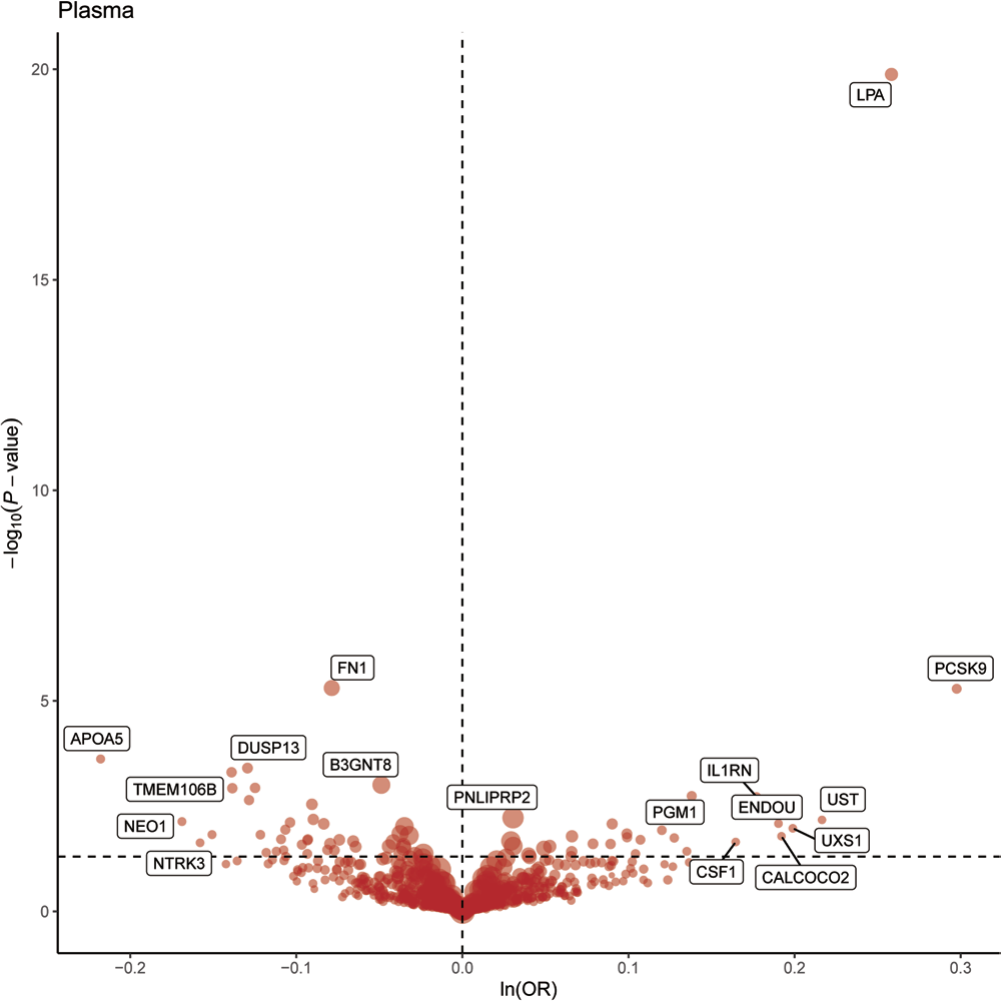
MR results for plasma proteins and the risk of MI. MI = myocardial infarction, MR = Mendelian randomization.

Under the condition of *P* < .01, the MR analysis revealed causal relationships between 22 proteins and MI (Fig. 2). Specifically, except for PNLIPRP2, RIDA, PGM1, IL1RN, ENDOU, UST, LPA, and PCSK9, an increase in the remaining 14 proteins (APOA5, NEO1, TGFB1, TMEM106B, SWAP70, B4GALT1, DUSP13, GPNMB, SERPINE2, EPHB2, FN1, PLG, B3GNT8, CPB2) was associated with a reduced incidence of MI. Detailed results are presented in Table 1.

**Table 1:** Information on MI-related proteins

Protein	SNP	OR (95% CI)	*P* value	F statistics	Author
SERPINE2	rs68066031	0.91 (0.86, 0.97)	2.86E-03	205.83	Sun
RIDA	rs1462977	1.09 (1.02, 1.17)	8.37E-03	172.34	Sun
CPB2	rs3742264	0.97 (0.94, 0.99)	9.57E-03	454.41	Suhre
APOA5	rs964184	0.80 (0.72, 0.90)	2.40E-04	52.29	Sun
SWAP70	rs415895	0.88 (0.81, 0.96)	2.28E-03	107.88	Sun
EPHB2	rs6687487	0.91 (0.86, 0.98)	6.54E-03	187.57	Sun
PGM1	rs1126728	1.15 (1.05, 1.25)	1.82E-03	98.55	Sun
FN1	rs1250258	0.92 (0.89, 0.96)	4.96E-06	245.58	Suhre
PLG	rs783150	0.92 (0.86, 0.98)	8.33E-03	62.08	Suhre
TGFB1	rs1800470	0.87 (0.80, 0.95)	1.19E-03	116.46	Emilsson
LPA	rs55730499	1.29 (1.25, 1.34)	1.32E-20	744.34	Yao
B4GALT1	rs7019909	0.88 (0.82, 0.95)	1.17E-03	124.08	Sun
IL1RN	rs6761276	1.19 (1.07, 1.33)	1.89E-03	59.13	Sun
ENDOU	rs2072117	1.21 (1.05, 1.39)	8.21E-03	39.3	Emilsson
PNLIPRP2	rs7910135	1.03 (1.01, 1.05)	5.97E-03	2898.03	Emilsson
GPNMB	rs2268748	0.90 (0.84, 0.97)	7.74E-03	123.11	Sun
NEO1	rs12903656	0.84 (0.75, 0.96)	7.36E-03	47.53	Sun
DUSP13	rs6480771	0.88 (0.82, 0.94)	3.95E-04	146.93	Sun
B3GNT8	rs284663	0.95 (0.93, 0.98)	9.91E-04	1255.18	Emilsson
PCSK9	rs191448950	1.35 (1.18, 1.53)	5.16E-06	89.49	Emilsson
TMEM106B	rs10950398	0.87 (0.80, 0.94)	4.96E-04	118.37	Emilsson
UST	rs11155591	1.24 (1.06, 1.45)	6.71E-03	32.84	Emilsson

MI = myocardial infarction, SNP = single nucleotide polymorphism.

### 3.2. Sensitivity analysis of MI-related proteins

Through additional co-localization analysis, we identified 2 of the 22 proteins associated with MI as potential drug targets, namely APOA5 and LPA. Firstly, the Steiger filtering was applied to ensure directionality further. Secondly, the Bayesian co-localization analysis strongly indicated that APOA5 (coloc.abf-PPH4 = 0.86) and LPA (coloc.abf-PPH4 = 1.00) shared the same genetic variants with MI.

### 3.3. External validation of potential drug targets in MI

Significant variant strategies were employed to replicate the main findings in different datasets. In the CARDIoGRAMplusC4D dataset, APOA5 (coloc.abf-PPH4 = 0.764) and LPA (coloc.abf-PPH4 = 1.000) exhibited the same variant concerning MI. Additionally, IL1RN (coloc.abf-PPH4 = 0.997) also shared the same variant. Similarly, the FinnGen study revealed that APOA5 (coloc.abf-PPH4 = 0.998) and LPA (coloc.abf-PPH4 = 1.000) had the same variant concerning MI. Moreover, FN1 (coloc.abf-PPH4 = 0.956), NT5C (coloc.abf-PPH4 = 0.934), and SEMA3C (coloc.abf-PPH4 = 0.835) displayed similar variants.

### 3.4. Relationship between potential drug targets and risk factors for heart disease

**Figure 3.**
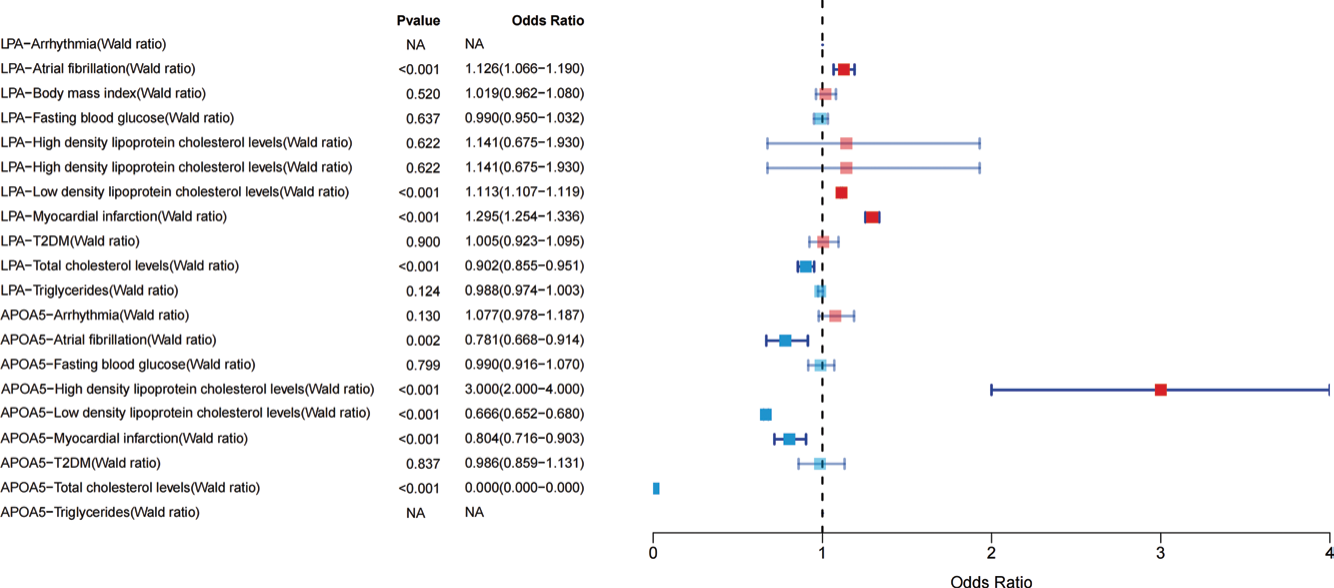
Relationship between potential drug targets and risk factors for heart disease.

Two potential drug targets, LPA and APOA5, only have a SNP. Therefore, MR analysis was performed using the Wald ratio. The results are as follows: an increase in LPA protein expression is a risk factor for atrial fibrillation, LDL, and MI but offers protection against TGs. On the contrary, an increase in APOA5 expression is protective against atrial fibrillation, HDL, MI, and total cholesterol but becomes a risk factor for LDL (Fig. 3).

### 3.5. PPI networks and enrichment analysis

**Figure 4.**
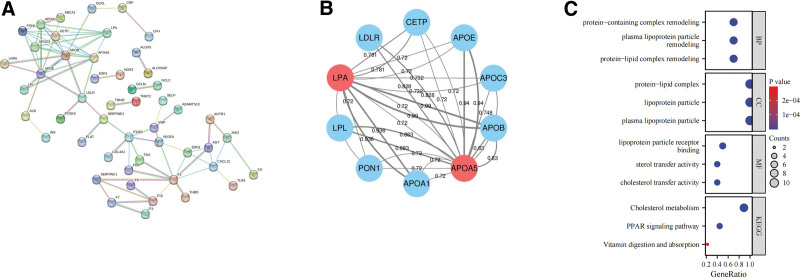
PPI Network and Enrichment analysis. (A) The PPI network based on MI-related targets. (B) The PPI network related to LPA and APOA5. (C) Enrichment analysis. MI = myocardial infarction, PPI = protein-protein interaction.

In the GeneCards database, 97 confirmed targets related to MI with a Score > 20 were obtained. Subsequently, LPA, APOA5, and the 97 screened targets were imported into the STRING database to hide unconnected nodes. This yielded a PPI network with 50 nodes and 83 edges (Fig. 4A). From this network, we extracted the targets associated with LPA and APOA5, including APOA1, APOB, APOC3, APOE, CETP, LDLR, LPL, and PON1 (Fig. 4B). Enrichment analysis revealed that these 10 nodes were mainly involved in biological processes such as protein-lipid complex remodeling, plasma lipoprotein particle remodeling, and protein-containing complex remodeling. They were also enriched in cellular components such as plasma lipoprotein particles, lipoprotein particles, and protein-lipid complexes. Additionally, they were enriched in molecular functions such as lipoprotein particle receptor binding, cholesterol transfer activity, and sterol transfer activity. Furthermore, these targets involved KEGG pathways such as cholesterol metabolism, peroxisome proliferator-activated receptor (PPAR) signaling pathway, and Vitamin digestion and absorption (Fig. 4C).

## 4. Discussion

MI, commonly known as a heart attack, occurs when plaque builds up in the arteries inner walls, leading to restricted blood flow and damage to the heart muscles due to insufficient oxygen supply.^[23]^ As one of the leading causes of death worldwide, there is an urgent need for novel therapeutic strategies to treat MI. In recent decades, therapeutic approaches for MI have focused on understanding the cellular and molecular processes underlying MI pathology and treatment. However, the current strategies, including pharmacotherapy, gene therapy, protein therapy, cell therapy, and exosome therapy, have limited clinical application prospects for MI recovery.^[24]^

This study represents the first attempt to utilize plasma proteomic data in exploring causal proteins for MI through 2-sample MR and Bayesian co-localization. We identified 2 proteins, LPA and APOA5, as potential drug targets for MI. Through MR, we observed that both plasma proteins shared the same variant with MI (PP.H4 > 0.75) and were externally validated in the CARDIoGRAMplusC4D and FinnGen databases, confirming our findings. LPA, an atherogenic lipoprotein, is genetically regulated, and its genetic variations are associated with increased levels of lipoprotein(a) (Lp(a)), which serves as a preferential carrier for oxidized phospholipids. Elevated Lp(a) levels have detrimental effects on endothelial function, inflammation, oxidative stress, and plaque stability, accelerating the formation of atherothrombotic plaques and serving as a key pathogenic factor in coronary artery disease.^[25,26]^ Notably, PCSK9 (proprotein convertase subtilisin/kexin 9) inhibitors have shown significant reductions in plasma Lp(a) concentration, which may lead to improved coronary outcomes.^[27]^ Pei et al^[28]^ have experimentally demonstrated the potential clinical therapeutic value of targeting LPA-LPA2 to protect the heart from ischemic injury, warranting further clinical attention.

Furthermore, APOA5 is believed to be the gene exerting the strongest influence on TG metabolism. Research studies have indicated that APOA5 variants affect the overall concentration of TG and impact the distribution of lipoprotein subclasses, leading to atherogenic dyslipidemia in high-risk individuals.^[29]^ In recent studies, coding sequence mutations in 2 genes associated with APOA5, namely lipoprotein lipase and apolipoprotein C3, have been connected to an increased risk of MI.^[30]^ Cosmin Tirdea further proposed that APOA5, one of the primary targets for increasing the risk of acute MI, exhibits a 2.2-fold higher risk for MI in individuals carrying rare missense mutations in the APOA5 promoter region. Hence, targeting APOA5 holds great promise as a therapeutic approach.^[31]^

Based on PPI and enrichment analysis, the mechanism of action for LPA and APOA5 appears to be linked to lipid metabolism. It has been demonstrated that lipid levels and composition in patients’ blood can predict MI and its associated complications.^[32]^ In a cohort study, the risk of MI was most accurately assessed by quantifying APOB-containing lipoproteins, implying that APOB may play a key role in atherosclerosis development. Therefore, therapeutic strategies should primarily reduce the concentration of all APOB-containing lipoproteins.^[33]^

The enrichment analysis results revealed that the KEGG pathway primarily focuses on cholesterol metabolism, the PPAR signaling pathway, and vitamin digestion and absorption. Among these pathways, PPARs are particularly important. PPARs belong to the nuclear hormone receptor family, acting as ligand-activated transcription factors, and exist in 3 isoforms: PPARα (NR1C1), PPARβ/δ(NR1C2), and PPARγ (NR1C3).^[34]^ Specifically, PPARβ/δis the predominant subtype expressed in cardiac tissue.^[35]^ Conditional deletion of PPARβ/δin cardiomyocytes has been associated with myocardial lipid accumulation and congestive heart failure with reduced survival. The primary role of PPARβ/δin maintaining normal fatty acid oxidation has been identified as the main mechanism for its cardioprotective action.^[36]^ In addition to its role in fatty acid oxidation, PPARs regulate various functions such as glucose and lipid homeostasis, inflammation, and development.

Activators of PPARs have been utilized to treat a range of metabolic disorders, including diabetes and hyperlipidemia, by activating specific PPAR isoforms. Blood lipids are well-established risk factors for MI. In a case-control study, lipoproteins and lipids showed similar associations with MI, where cholesterol concentrations within HDL particles were inversely associated, while TG concentrations were positively associated with MI.^[37]^ Current cholesterol guidelines prioritize LDL cholesterol as the primary target, followed by APOB and non-HDL cholesterol as secondary targets. A Copenhagen General Population Study involving 13,015 statin-treated patients demonstrated that elevated APOB and non-HDL cholesterol, but not LDL cholesterol, were associated with an increased risk of all-cause mortality and MI.^[38]^ Moreover, APOB was a more accurate marker for the risk of MI than LDL cholesterol. The relationship between metabolic pathways and MI has received considerable attention. A comprehensive analysis incorporating metabolomics and proteomics revealed that myocardial injury following MI is closely associated with several metabolic pathways, particularly those involved in energy metabolism, amino acid metabolism, vascular smooth muscle contraction, gap junctions, and neuroactive ligand-receptor interactions.^[39]^ These findings contribute to a better understanding of the underlying mechanisms of MI and have implications for identifying potential therapeutic targets.

Cholesterol metabolism is an important pathway for MI. HMGCR is an important rate-limiting enzyme in the cholesterol synthesis pathway, and its activity is regulated, which affects cholesterol synthesis. Studies have shown that the occurrence and development of cardiovascular diseases can be influenced by regulating cholesterol metabolism pathways.^[40]^ For example, statin drugs (such as atorvastatin) can inhibit the activity of HMGCR, thereby reducing cholesterol synthesis, lowering LDL cholesterol levels in the blood, and reducing the risk of cardiovascular diseases. Additionally, some drugs and compounds can also affect the transport and clearance of cholesterol by regulating the expression and function of LDL-R. By regulating cholesterol metabolism pathways, cholesterol levels can be lowered, reducing the risk of cardiovascular diseases.

PPAR is a type of nuclear receptor that is involved in regulating physiological processes such as lipid metabolism and inflammation. In MI, the activity of the PPAR pathway undergoes changes, which have an impact on the development and prognosis of MI. PPAR-α is a member of the PPAR family, studies^[41,42]^ have shown that its activation can inhibit inflammatory response and oxidative stress, reduce myocardial injury in MI, promote metabolic adaptation of myocardial cells, enhance the antioxidant capacity of myocardial cells, and reduce apoptosis and necrosis of myocardial cells. Additionally, PPAR-γ also participates in regulating the development of MI,^[43]^ as it can inhibit inflammatory response and fibrosis process, reduce cardiac remodeling after MI, promote survival and repair of myocardial cells, and improve cardiac function after MI. In summary, the PPAR pathway plays an important role in MI, and its activation can inhibit inflammatory response, oxidative stress, and fibrosis process, alleviate myocardial damage, and promote survival and repair of myocardial cells. Modulating the activity of the PPAR pathway may be a potential strategy for the treatment of MI.

In summary, our comprehensive analysis demonstrates a causal relationship between genetically determined circulating LPA and APOA5 levels and the risk of MI. The identified proteins, particularly LPA and APOA5, may serve as attractive drug targets for MI. Further research is needed to explore the role of these candidate proteins in MI. Moreover, IL1RN, FN1, NT5C, and SEMA3C appear to have potential as drug targets, but more studies are required to confirm their therapeutic value due to the low level of evidence. It is important to acknowledge the limitations of this study. Firstly, most of the proteins only showed only one cis-acting pQTLs and lacked trans-acting pQTLs, limiting the analysis, including MR-Egger, heterogeneity tests, and multitest correction. Additionally, although this study verified the findings using multiple datasets, the generalizability is limited as these datasets primarily focus on European populations, lacking validation in other populations.

## Author contributions

**Conceptualization:** Jiayu Wu, Xianqiong Zhu.

**Formal analysis:** Qi He.

**Investigation:** Jiayu Wu, Xianqiong Zhu, Qiaoming Fan, Huilian Cai, Ying Xu.

**Methodology:** Qiaoming Fan, Ying Xu, Xiaolin He, Yan Li.

**Supervision:** Xingwei Di.

**Validation:** Jiayu Wu.

**Writing – original draft:** Xianqiong Zhu, Qi He.

**Writing – review & editing:** Jiayu Wu, Qiaoming Fan, Ying Xu.
